# Carfilzomib enhances natural killer cell-mediated lysis of myeloma linked with decreasing expression of HLA class I

**DOI:** 10.18632/oncotarget.4831

**Published:** 2015-08-20

**Authors:** Guang Yang, Minjie Gao, Yiwen Zhang, Yuanyuan Kong, Lu Gao, Yi Tao, Ying Han, Huiqun Wu, Xiuqin Meng, Hongwei Xu, Fenghuang Zhan, Xiaosong Wu, Jumei Shi

**Affiliations:** ^1^ Department of Hematology, Shanghai Tenth People's Hospital, Tongji University School of Medicine, Shanghai, China; ^2^ Department of Internal Medicine, University of Iowa, Carver College of Medicine, Iowa City, Iowa, USA

**Keywords:** carfilzomib, multiple myeloma, natural killer cell, proteasome inhibitor, histocompatibility antigens class I

## Abstract

Natural killer (NK) cell-based treatments are promising therapies for multiple myeloma (MM). Carfilzomib (CFZ), is a second-generation proteasome inhibitor, used to treat relapsed and refractory MM. In this study, we determined that CFZ treatment enhanced the sensitivity of MM cells to NK cell-mediated lysis. Here, we report that CFZ decreased the expression of human leukocyte antigen (HLA) class I in a time- and dose- dependent manner. CFZ also down-regulated the expression of newly formed HLA class I on MM cells. Treatment of MM with CFZ enhanced NK cell degranulation and significantly sensitized patients' MM cells to NK cell-mediated lysis. Furthermore, the enhancement of NK cell-mediated lysis was linked with the decreased expression of HLA class I. Our findings show a novel activity of CFZ as an immunomodulating agent and suggest a possible approach to therapeutically augment NK cell function in MM patients.

## INTRODUCTION

Multiple myeloma (MM) is a malignant disorder characterized by uncontrolled monoclonal plasma cell proliferation [1]. It accounts for 10% of all hematological malignancies and causes 15–20% of deaths from hematological malignancies. Although new therapies were introduced and overall survival of MM was improved in the last 10 years, MM still remains an incurable disease due to drug resistance [2, 3].

Allogeneic stem cell transplantation (Allo-SCT) improves long-term survival in MM patients [4], but Allo-SCT in MM is still a controversial treatment [5]. The human natural killer (NK) cell role in killing tumor cells is suggested by the effects of Allo-SCT, when the grafted NK inhibitory receptors mismatch with recipient HLA molecules [6]. NK cells are a subpopulation of lymphocytes that play an important role in both innate immunity and adaptive immunity [7]. NK cell-mediated cytotoxicity is enhanced or blocked by a balance between activating and inhibitory signals sent by membrane receptors [8]. The interactions between inhibitory killer cell immunoglobulin-like receptor (KIR) and self HLA class I render NK cells tolerant to self, a process known as “NK cell licensing”. So mature NK cells will not attack healthy cells [9]. Down-regulation of the expression of HLA class I on tumor cells helps them escape immune surveillance. However, according to the “missing self-hypothesis”, lack of normal expression of HLA class I will activate NK cells, so they can recognize and lyse HLA class I defective tumor cells [10].

HLA class I expressed on the cell surface consist of two polypeptide chains: a heavy chain (α chain) and a light chain (β2M), and an additional peptide of 8 to 10 amino acids in length [11]. Binding to the peptide is a requirement for class I assembly in the endoplasmic reticulum [12]. Stabilization of the HLA class I complex on the cell surface results from this peptide binding [13]. The human T2 cell line, that has a defect in transporting peptides from the cytosol into the endoplasmic reticulum, was reported to be HLA class I assembly deficient [14]. Proteasome is believed to be the cytosolic factor responsible for peptide generation [15]. Proteasome inhibitor can block the assembly of HLA class I by inhibiting the peptide production [16].

Carfilzomib (CFZ), a selective proteasome inhibitor, is used to treat patients with MM who are refractory or intolerant to both bortezomib and lenalidomide (or thalidomide) [17]. CFZ irreversibly inhibits 26S proteasome binding, causing sustained proteasome inhibition [18]. We hypothesized that CFZ, a second-generation proteasome inhibitor, could decrease the expression of HLA class I on MM cells. We also investigated whether CFZ treatment of MM cells could enhance the lysis mediated by NK cells, thus linking this process with the down-regulation of HLA expression. Herein, we show a novel activity of CFZ as an immunomodulator, and suggest its use as a possible drug therapeutic approach to combine with NK cell-based therapies for MM.

## RESULTS

### CFZ decreases the expression of HLA-ABC in MM cell lines and in cells from MM patients

**Figure 1:**
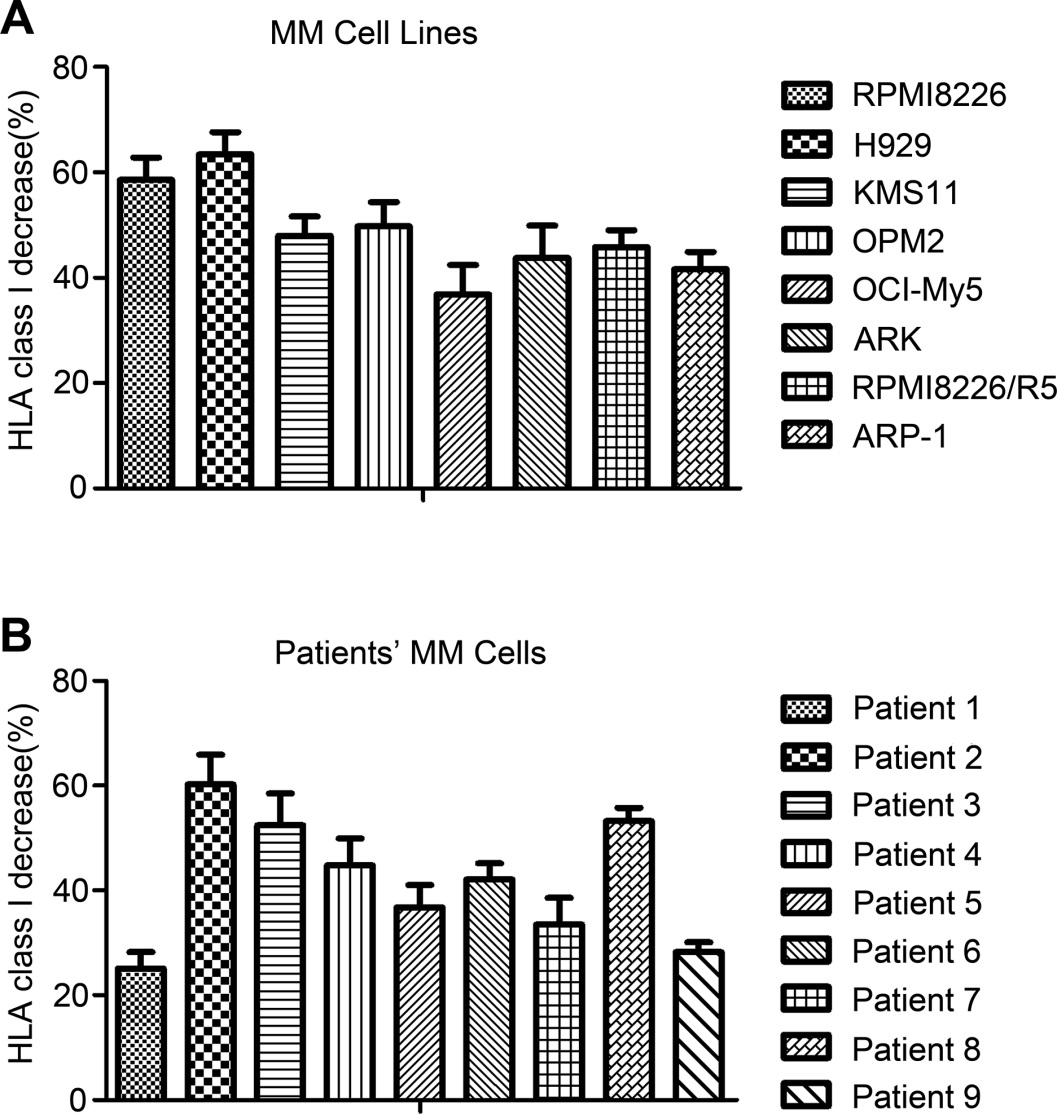
Expression of HLA class I decreased after CFZ treatment in MM cell lines and primary MM cells **A.** MM cells were incubated with 10 nM CFZ for 24 hours, then cells were stained with FITC-HLA-ABC, APC-Annexin V and 7AAD. Flow cytometer was used to gate the both Annexin V and 7AAD double negative cells and the mean-fluorescence intensity (MFI) was recorded. Class I decrease % = 100 × (MFI of control - MFI of treated cells)/MFI of control. **B.** The patients' MM cells were treated with 20 to 40 nM CFZ for 24 hours. MFI was recorded to test the down-regulation of HLA.

To study the impact of CFZ treatment on the expression of HLA on MM cells, we used flow cytometric analysis to test MM cell lines (KMS11, H929, RPMI8226, OCI-MY5, OPM2, ARK, U266, ARP-1, and RPMI8226/R5) by gating annexin V and 7AAD double-negative cells. As shown in Figure 1A, the expression of HLA class I decreased after 10 nM CFZ treatment, including the bortezomib resistant cell line RPMI8226/R5. The mean reduction was 47.7 ± 9.4%. In Figure 1B, we showed that 20 to 40 nM CFZ, used to treat the patients' MM cells, down-regulated HLA class I with a mean reduction of 42.8 ± 12.4% (*n* = 9). CFZ induced apoptosis in myeloma cells is shown in Supplementary Figure S1A and S1B. We also used CFZ to treat other cancer cell types (one renal cell carcinoma and two breast cancer cell lines) and normal cells (CD34+ cells and monocytes), but down-regulation of HLA class I was not observed (data not shown). These results suggest the specificity of CFZ induce down-regulation of HLA class I expression on myeloma cells.

**Figure 2:**
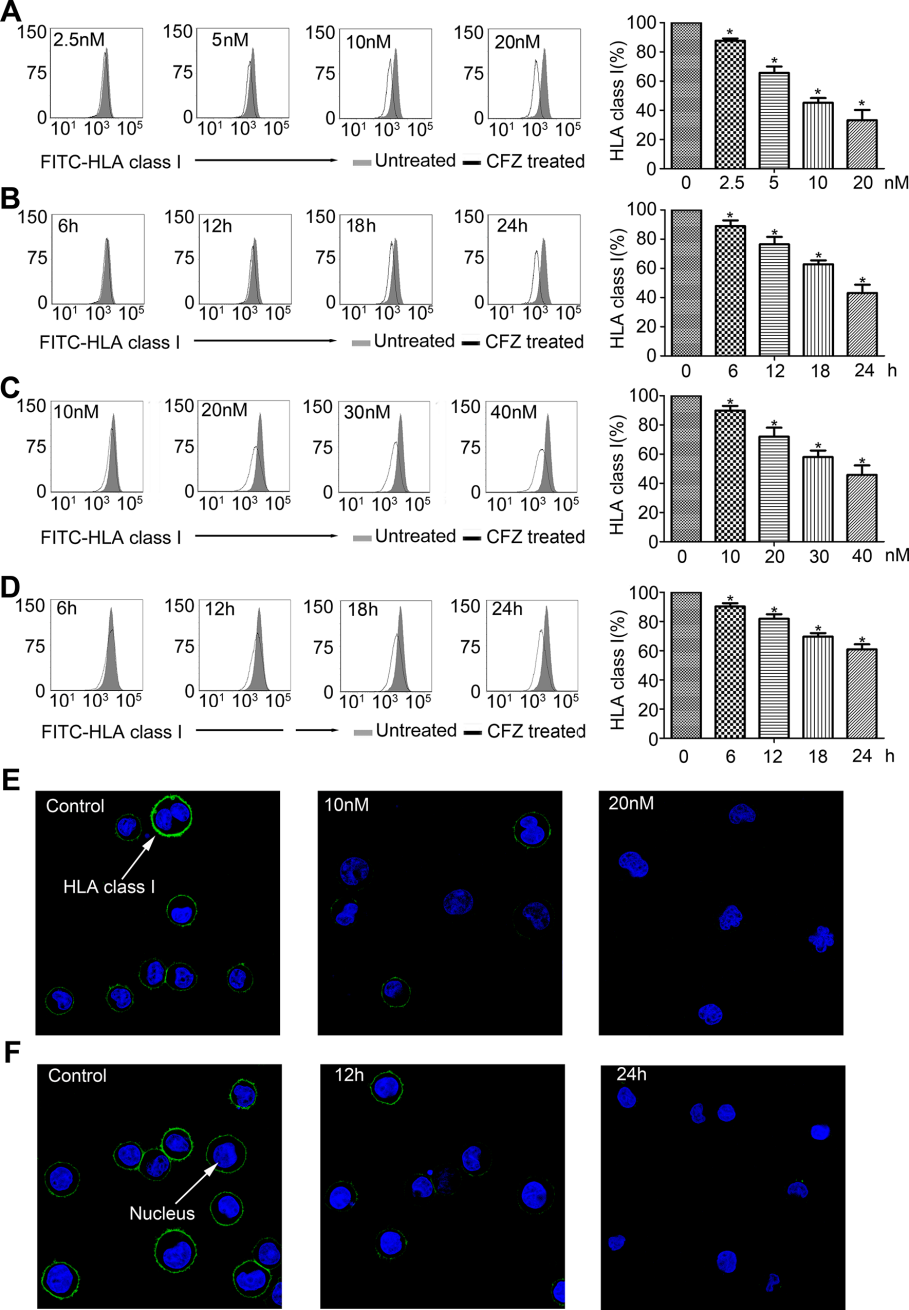
Down-regulation of HLA class I was in a dose- and time-dependent manner **A.** H929 was treated with different doses of CFZ for 24 hours. **B.** H929 was treated with 10 nM CFZ for different durations. **C.** Primary MM cells were treated with different doses of CFZ for 24 hours. **D.** Primary MM cells were treated with 40 nM CFZ for different durations. **E.** and **F.** Immunofluorescence analysis was performed to confirm the consequence that down-regulation of HLA class I was in a dose- and time-dependent manner. *P < 0.05.

We then used different concentrations of CFZ or different durations of CFZ treatment on the H929 cell line. We found that down-regulation of HLA class I expression was in a dose- and time-dependent manner (Figure 2A and 2B). These results were confirmed by using immunofluorescence analyses (Figure 2E and 2F). The kinetics analyses of apoptosis after CFZ treatment are presented in Supplementary Figure S1C and S1D. Similar results were obtained in primary MM cells (Figure 2C and 2D).

HLA-C is a more specialized ligand for KIRs, as compared to HLA-A and -B, approximately the same level of down-regulation of HLA-C was obtained after CFZ treatment (data not shown). Then we investigated whether the exogenous HLA-C binding peptides (mentioned in Materials and Methods) could rescue the down-regulation of HLA-C caused by CFZ. The expression level of HLA-C and HLA class I remained almost unchanged in the presence of the peptides and Human β2M cocultured with the CFZ treated H929 cells (Supplementary Figure S2). The peptides had no effect on the HLA-C and HLA class I expression levels in the untreated H929 cells (Supplementary Figure S2). These data indicate that exogenous HLA-C binding peptides can stabilize HLA-C expression on the cell surface during CFZ treatment.

**Figure 3:**
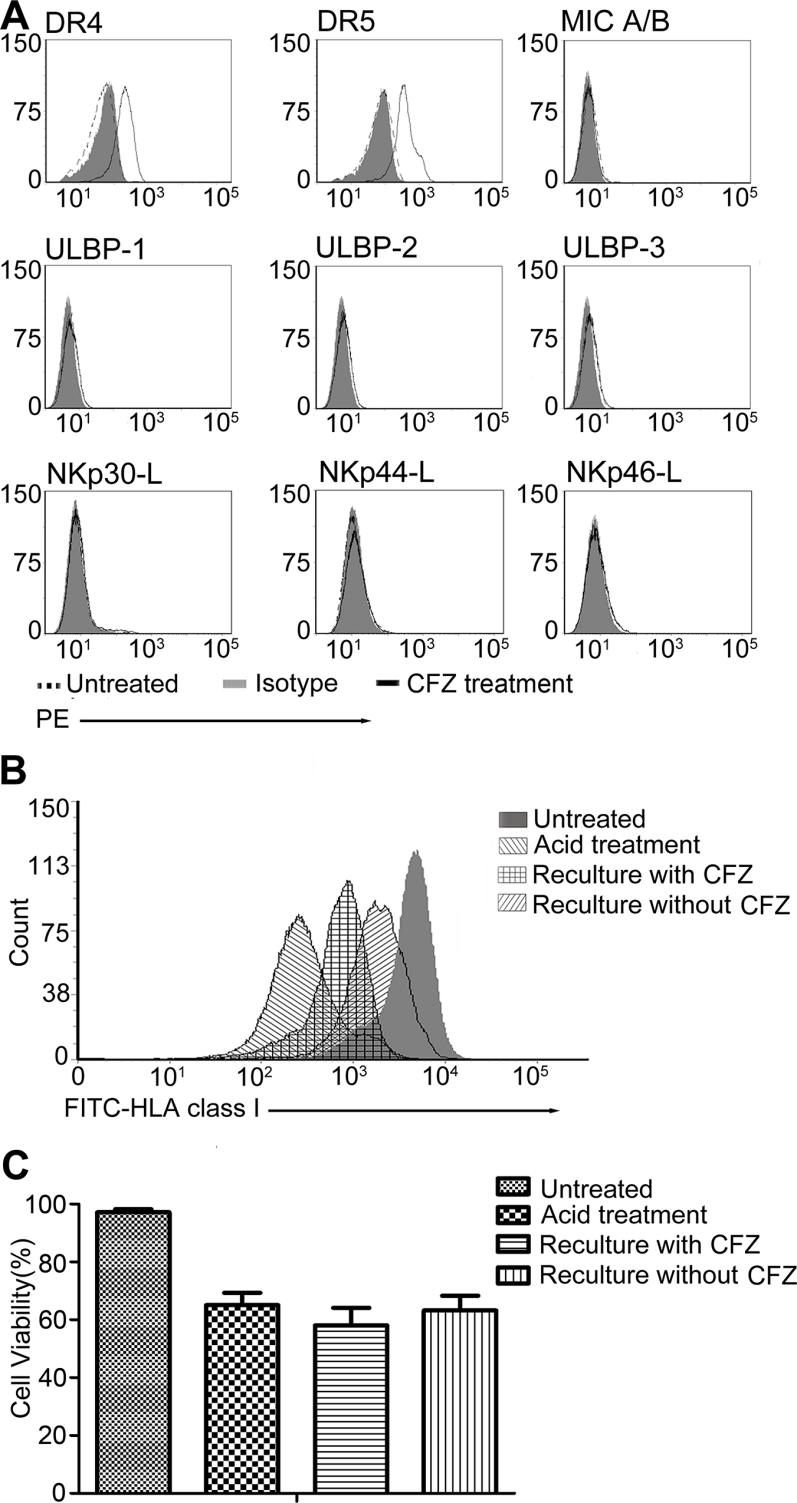
CFZ up-regulated DR4, DR5 and affected the re-expression of HLA class I on cell surface, but had no effect on ULBP 1–3, MIC A/B, NKp30-L, NKp44-L and NKp46-L **A.** H929 was treated with 10 nM CFZ for 24 hours. Flow cytometer was used to detect the expression of DR4, DR5, ULBP1–3, MIC A/B, NKp30-L, NKp44-L and NKp46-L. MFI of DR4 and DR5 were increased after CFZ treatment (DR4: 195.3 ± 6.1 vs 44.1 ± 2.6 and DR5: 363.2 ± 9.2 vs 79.3 ± 3.8) **B.** Acid stripping was performed to remove the HLA class I on H929 cell surface as described in the Material and Methods, then cells were recultured with or without CFZ for 12 hours to investigate the re-expression of HLA class I. **C.** The survival of H929 after acid treatment was 65.1% ± 4.3, compared to the untreated group (97.1% ± 1.1). The viability of H929 was similar in the presence (58.6% ± 6.1) or absence (63.2% ± 5.1) of CFZ.

We also determined the expression levels of other NK cell ligands on H929 cells after CFZ treatment, as shown in Figure 3A, CFZ could up-regulate the expression of DR4 and DR5, but had no effect on the ligands of NKG2D (MIC A/B, ULBP 1–3) and ligands of NCRs (NKp30-L, NKp44-L and NKp46-L).

### CFZ decreases the amount of newly formed HLA class I on the MM cell surface

We have shown the down-regulation of HLA class I expression on MM cells after CFZ treatment. We next used acid stripping, which proved to selectively measure the surface expression of newly formed class I complex, to investigate whether CFZ had an effect on the generation of new HLA class I. H929 cells were exposed to glycine (pH = 2.5) to remove surface HLA class I, and then allowed to re-express newly generated class I in the presence or absence of 10 nM CFZ for 12 hours. The expression of HLA class I is presented in Figure 3B. After treatment of H929 cells with acid, the expression of HLA class I (MFI = 279) was nearly undetectable by flow cytometry. In contrast, the MFI of untreated cells was 4295. In the absence of CFZ, the expression of HLA class I (MFI = 1848) was observed in nearly 40% of the untreated cells, and was much lower after CFZ treatment for 12 hours (MFI = 762, only 17% of the untreated cells). The proportion of live cells in the presence or absence of CFZ is shown in Figure 3C. Taken together, these data show that the expression of newly generated HLA class I is inhibited by CFZ.

**Figure 4:**
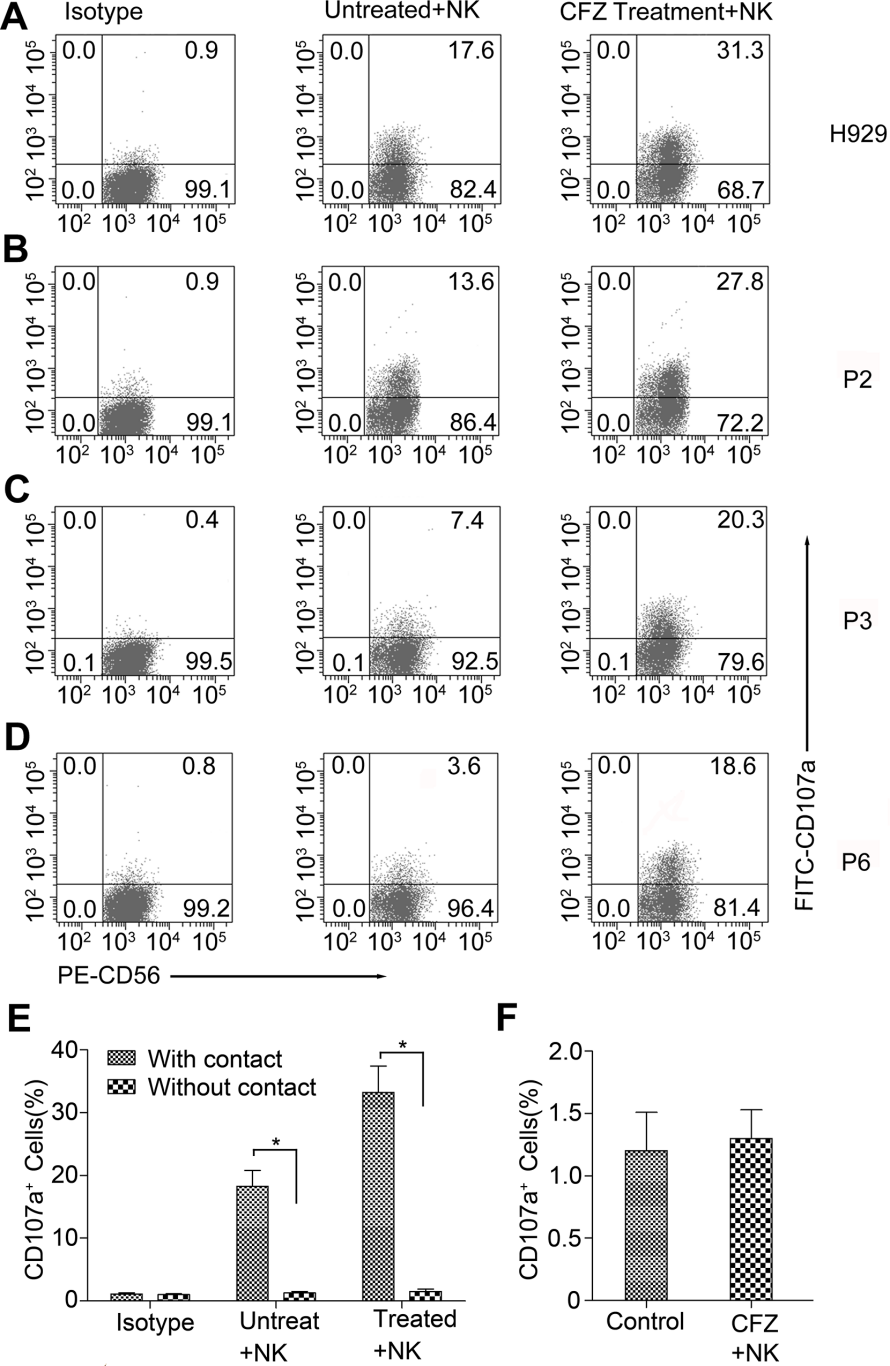
CFZ treatment enhanced the NK cell degranulation in MM cell lines and patients' MM cells **A–D.** MM cells were cultured in the presence or absence of CFZ for 24 hours. We used 10 nM CFZ to treat MM cell line H929 and 20 to 40 nM CFZ to treat patients' MM cells. Then both the treated or untreated cells were cocultured with NK cells at an E/T ratio of 5:1 for 4 hours. FITC-conjugated CD107a was added at the beginning of the culture and monensin was added at the second hour of culture. After that cells were washed and stained with PE-conjugated CD56 for 30 minutes. Flow cytometer was used to analyze the expression of CD107a on NK cells. B-D represented three of the nine patients' MM cells. P represented Patient. *P < 0.05. **E.** CFZ treated or untreated H929 were cocultured with or without the contact of NK cells at an E/T ratio of 5:1 for 4 hours. **F.** NK cells were treated with CFZ for 4 hours to detect the expression of CD107a.

### NK cell degranulation is enhanced by CFZ treatment on myeloma cells

Our previous studies showed that CFZ could decrease the expression and re-expression levels of HLA class I on the MM cell surface. To investigate whether CFZ treatment could activate NK cell functions, a CD107a degranulation assay was used, and CD107a expression levels were used to measure NK-cell degranulation [19]. NK cells were incubated with CFZ treated or untreated MM cells and stained with FITC-conjugated CD107a and PE-conjugated CD56. Figure 4A–4D show us representative degranulation assays. When NK cells were incubated with H929 cells, without CFZ, only a small percent of CD107a^+^ NK cells were obtained (Figure 4A). In contrast, when NK cells were incubated with MM cells with 10 nM CFZ, the percentage of NK cells expressing CD107a on the surface greatly increased (mean ± SD: 33.6 ± 2.1%, for treated cells vs 16.7 ± 2.3%, for control cells, *P* < 0.05). Similar results were obtained when MM patients' cells were exposed to 20 to 40 nM CFZ (mean ± SD: 23.6 ± 3.1% vs 7.6 ± 3.0%, *n* = 9, *P* < 0.01). Figure 4B–4D represents three of the nine patients' MM samples. Next we used CFZ treated H929 cells to coculture with or without the contact of NK cell, Figure 4E shows that the activation of NK cells requires MM/NK cell-cell contact. We also found that CFZ could not directly activated NK cells (Figure 4F). These findings suggest that CFZ treated MM cells can enhance NK cell degranulation.

### CFZ treatment sensitizes MM cells to NK cell-mediated lysis

**Figure 5:**
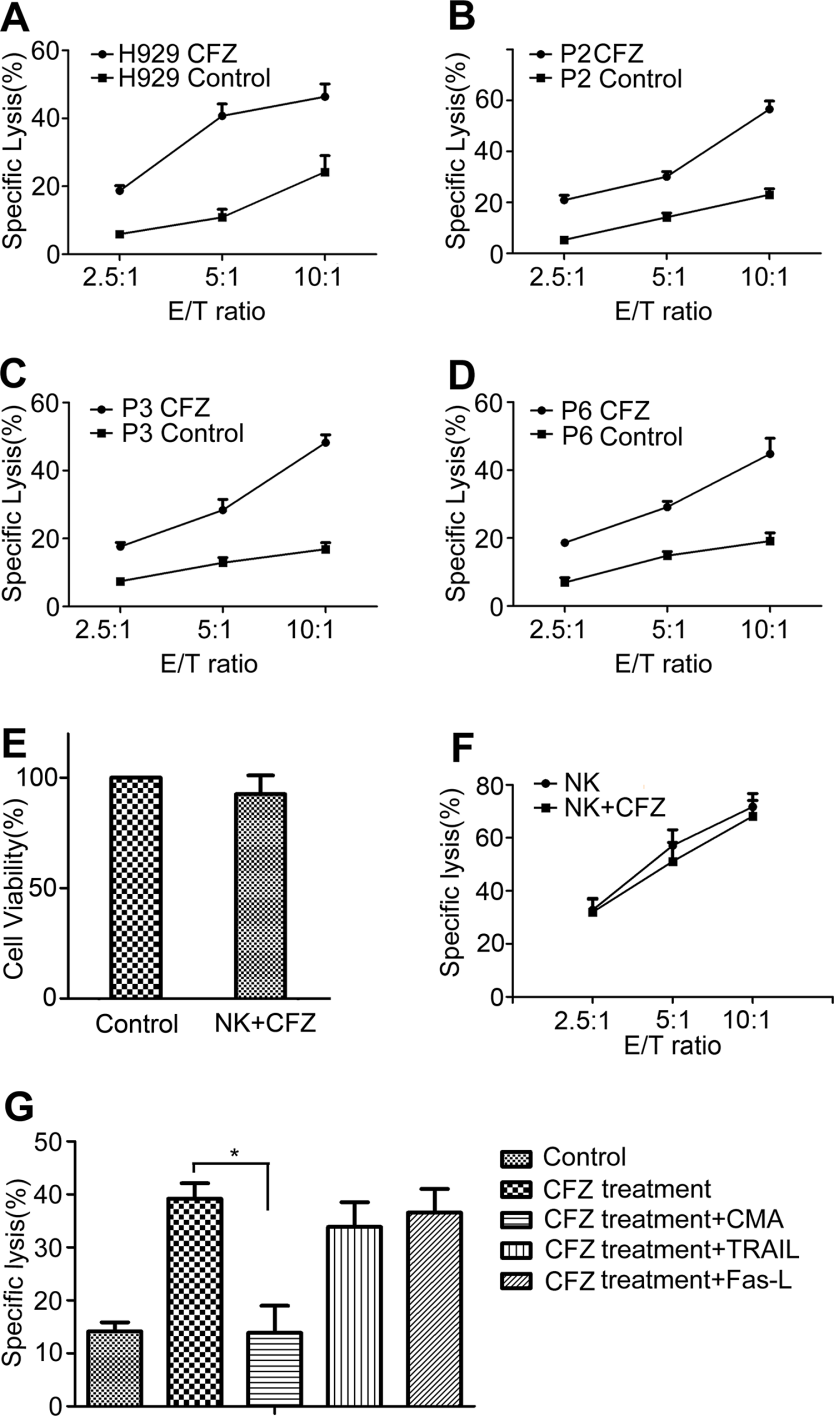
CFZ treatment enhanced NK cell-mediated lysis **A–D.** H929 was treated with 10 nM CFZ for 24 hours. Patients' MM cells were exposed to 20 to 40 nM CFZ for 24 hours. After they were stained with 100 μCi ^51^Cr for 1 hour, cells were cocultured with NK cells at different E/T ratio for 4 hours. Specific lysis percentage was calculated as (experimental release − spontaneous release) / (maximal release − spontaneous release) × 100. B-D represented three of the nine patients' MM cells. P represented Patient. **E.** NK cells were cultured in the presence or absence of CFZ for 12 hours. Then cells were collected and stained with FITC-annexin V and PI. Flow cytometer was used to gate annexin V and PI double negative cells. Untreated NK cells were defined as the control group. **F.** NK cells were cultured in the presence or absence of CFZ for 12 hours. ^51^Cr release assay (mentioned above) was used to analysis the NK cell-mediated lysis. The NK cell-sensitive cell line K562 was used as the target cells. **G.** CMA (100 nM), anti–Fas-L (5 μg/ml), and anti-TRAIL mAb (2.5 μg/ml) were used to test the cytotoxic pathways used by NK cells at an E/T ratio of 5:1. *P < 0.05.

CFZ treatment decreased the HLA class I expression on MM cells and enhanced NK cell degranulation. ^51^Cr release assays were used to evaluate NK cell-mediated lysis. H929 cells were treated with 10 nM CFZ and MM patients' cells were treated with 20 to 40 nM CFZ. As shown in Figure 5A, after treatment of H929 cells with CFZ, the cells showed higher sensitivity to NK cell-mediated lysis than untreated H929 cells (mean ± SD: 46.4 ± 3.7% vs 24.2 ± 4.8% at E/T 10:1, *P* < 0.01). CFZ also significantly enhanced the sensitivity of patients' MM cells to NK cell-mediated lysis (mean ± SD: 43.1 ± 6.4% vs 16.1 ± 4.0% at E/T 10:1, *n* = 9, *P* < 0.01). Figure 5B–5D represents three of the nine patients' MM cell samples. When using CFZ to treat NK cells, the viability and cytotoxicity of NK cells were not affected (Figure 5E and 5F).

We found that CFZ treatment enhanced NK cell degranulation. In order to identify the cytotoxic pathway used by NK cells, blocking experiments were performed. The antagonistic antibodies TRAIL and Fas-L had no effect on the cytotoxicity of NK cell. However, perforin/granzyme inhibitor CMA prevented the enhancement of NK cell–mediated lysis in the CFZ treated samples (Figure 5G), indicating that NK cell killing of MM cells after CFZ treatment was mediated by perforin/granzyme.

### Enhancement of NK cell-mediated lysis by CFZ treatment is associated with a decrease of HLA class I

**Figure 6:**
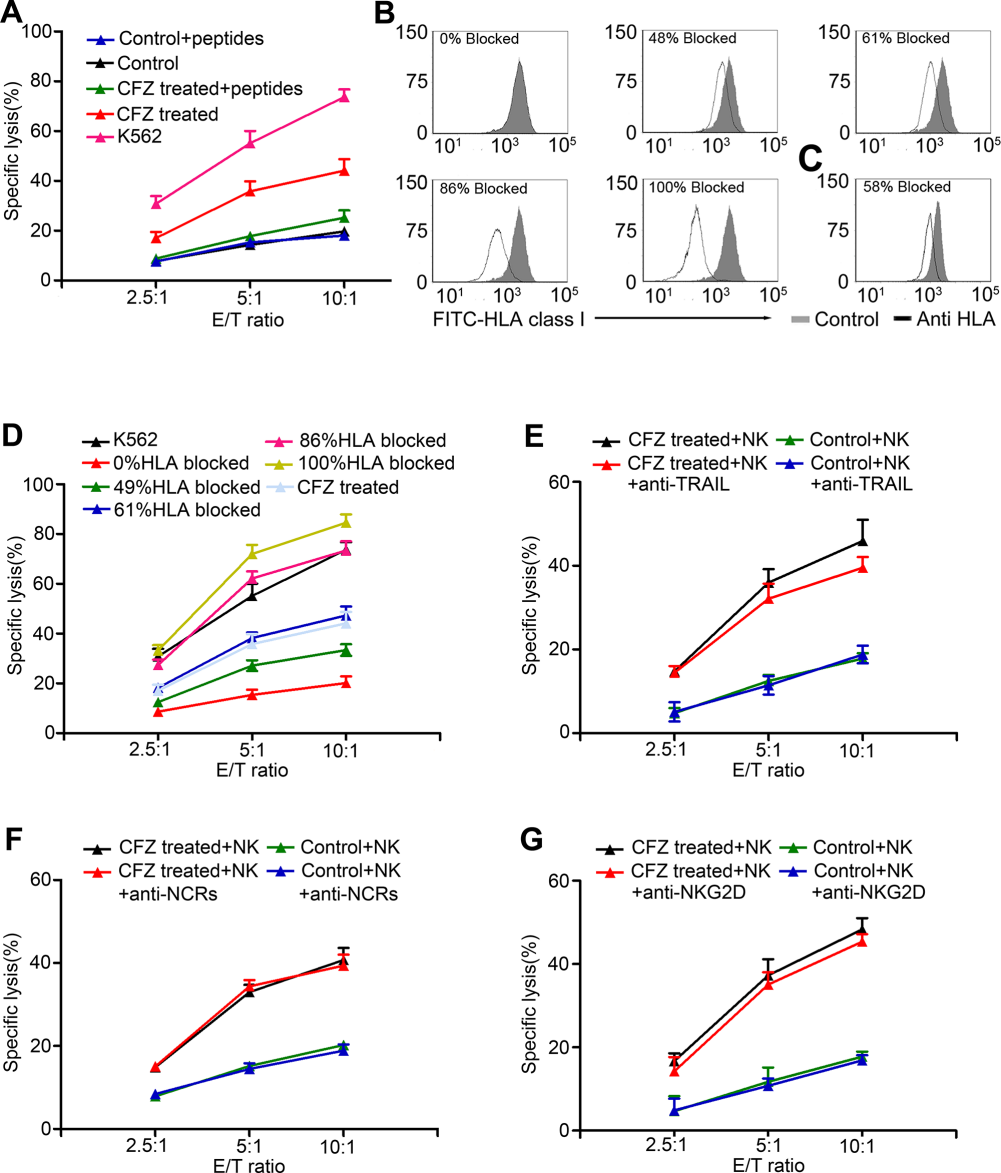
Enhancement of NK cell-mediated lysis was associated with down-regulation of HLA class I **A.** Exogenous binding peptides and Human β2M were added when H929 were treated by CFZ. ^51^Cr release assay was performed to analysis the NK cell-mediated lysis at different E/T ratio. **B.** Different concentrations of anti-HLA antibody were used to block the expression of HLA on H929 surface. **C.** 10 nM CFZ treatment for 24 hours caused 58% reduction in HLA class I expression. **D.** NK cell-mediated lysis was enhanced by the increasing percentage of HLA blocking. **E.** The enhancement of NK cell-mediated lysis was not mainly affected by the blocking of TRAIL on NK cells. **F.** The enhancement of NK cell-mediated lysis was not mainly affected by the blocking of NCRs on NK cells. **G.** The enhancement of NK cell-mediated lysis was not mainly affected by the blocking of NKG2D on NK cells. The E/T ratio were 2.5:1, 5:1, and 10:1.

We previously found that exogenous HLA-C binding peptides could rescue the down-regulation of HLA-C during CFZ treatment. To determine if NK cell cytotoxicity was linked with HLA decreases, cytotoxicity experiments were used. When exogenous HLA-C binding peptides and human β2M were added to the CFZ treated group, specific lysis was reduced to similar levels as the untreated group (Figure 6A). These results supported the hypothesis that enhancement of NK-cell mediated lysis by CFZ was linked with the reduction of HLA class I.

We next investigated if the down-regulation of HLA caused by CFZ was not an epiphenomenon but was physiologically relevant. Different concentrations of HLA-ABC antibody was used to block the HLA class I on the H929 cell line (Figure 6B) and 10 nM CFZ treatment could block 58% HLA class I expression (Figure 6C). A cytotoxicity assay was then used to evaluate NK cell-mediated lysis. As shown in Figure 6D, increasing concentrations of HLA antibody led to enhanced NK cell-mediated lysis. The lysis curves are almost identical between the CFZ treated MM cells and the 61% HLA blocked MM cells. These results indicated that HLA class I reduction caused by CFZ is physiologically relevant and was compatible with the enhancement of NK cell cytotoxicity.

We also found that CFZ up-regulated DR4 and DR5 expression on MM cells, which could enhance the NK cell killing via the TRAIL pathway. We used anti-TRAIL mAb in CFZ treated H929 cells. As shown in Figure 6E, the level of NK cell-mediated lysis was not affected by blocking TRAIL. The data suggest that CFZ has no effect on MIC A/B, ULBP 1–3, NKp30-L, NKp44-L, and NKp46-L (Figure 3A), which are the ligands responsible for activating receptors NKG2D and NCRs on NK cells. After using mAb to block NCRs and NKG2D, the levels of NK cell-mediated lysis in H929 cells were almost the same (Figure 6F and 6G). Similar results were obtained when we repeated these blocking experiments with primary human MM cells (Supplementary Figure S3A–S3C).

## DISCUSSION

Inhibition of the proteasome can block the degradation of misfolded proteins and induce cell apoptosis [20]. The high level of proteasome activity in tumor cells suggests the use of proteasome inhibitor as therapeutic agents against MM [21]. The first-in-class proteasome inhibitor bortezomib renovated the treatment of MM due to its anti-myeloma activity both in preclinical and clinical studies [22–24]. CFZ, a second-generation proteasome inhibitor which can overcome bortezomib resistance, is used for treatment of relapsed and refractory MM [25]. Proteasome also plays a role in processing of the peptide necessary for MHC class I mediated antigen presentation [26]. Through affecting such peptide generation, inhibition of proteasome will block class I assembly [27]. In this study, we found that CFZ could decrease the expression of HLA class I in both MM cell lines and patients' MM cells. The down-regulation caused by CFZ occurred in a dose- and time-dependent manner. These results were confirmed by immunofluorescence assays. Furthermore, we compared the cell surface levels of HLA class I on MM cells in the presence or absence of CFZ after acid treatment. We found that CFZ could inhibit the re-expression of HLA class I. The results using CFZ suggest that the proteasome is crucial for the generation of peptide to assemble class I. Our findings were in agreement with previous studies [15, 28, 29]. We also found that CFZ treatment could enhance the NK cell degranulation both in MM cell lines and patients' MM cells. In addition to perforin/granzyme-mediated cytotoxity, NK cells also expressed Fas-L and TRAIL to increase tumor cells killings. We also showed that CFZ augmented NK-cell cytotoxity by a perforin/granzyme-mediated mechanism, because such enhancement was abolished when CMA, but not anti-TRAIL or anti-Fas-L antibodies, was added.

Studies have shown that haplo-identical killer immunoglobulin-like receptor (KIR)-ligand (KIR-L) mismatched NK cells can kill chemo-resistant myeloma cells [30]. Although this treatment modality can be safely given to high-risk MM patients, alloreactive NK cell transfusion cannot always be achieved. This results in inhibition of most NK cells by HLA class I of patients [31]. We showed that CFZ treatment could down-regulate the expression of HLA class I on MM cells, and could sensitize MM cells to NK cell-mediated lysis. The doses of CFZ, used to treat MM cells, did not affect the NK-cell viability and function, implying that CFZ might be useful as a NK cell based therapy for MM.

In the cytotoxicity experiment, the exogenous HLA-C binding peptides, used in the CFZ treated group rescued the down-regulation of HLA-C and reduced NK cell-mediated lysis to a similar level as in the untreated group. This implied that the enhancement of NK cell cytotoxicity was linked with the down-regulation of HLA class I caused by CFZ. Such enhancement is demonstrated by the similar lysis curve, observed when HLA class I was blocked by using anti-HLA antibody. These results support our hypothesis that reduction of HLA class I in CFZ treated cells is physiologically relevant.

The expression of receptors on NK cell surfaces can either stimulate NK cell reactivity or suppress NK cell reactivity, and the individual receptors are known as activating receptors and inhibitory receptors, respectively [32]. In humans, KIR is one of the most important inhibitory receptors for HLA class as it governs the capacity of NK cells to display potent cytotoxicity to cells with low expression of HLA class I on the cell surface [33]. In addition to KIR, other activating receptors on NK cells, such as NKG2D, and the NCRs, NKp46/NCR1, NKp30/NCR3, and NKp44/NCR2 can also regulate NK cell function [34, 35]. MIC A/B and ULBP1-3 are the two groups of NKG2D ligands in humans. NCRs are among the first identified activating receptors on human NK cells. NCRs recognize ligands on a large array of tumor cells [36], although their physiological ligands remain unknown [35]. In our study, CFZ treatment had no effect on the expression of ligands of NKG2D or on NCRs. Blocking NKG2D and NCRs did not have a significant impact on NK cell lysis of myeloma cells. Previous studies showed that proteasome inhibitor could upregulate the TRAIL receptors on tumor cells [37–39]. Our studies showed that CFZ treatment could up-regulate DR4 and DR5 expression on MM cell surface, but NK cell-mediated lysis was not decreased when TRAIL was blocked. Taken together, our data show that CFZ augments the NK cell-mediated cell lysis, mainly by decreasing the expression of HLA class I on MM cell surfaces.

In the present study, we have shown that CFZ treatment of MM cells down-regulates the HLA class I expression and enhances NK cell-mediated lysis. Our study suggests a novel activity of CFZ as an immunomodulating agent. In the future, CFZ treatment combined with NK cell based therapies could potentially enhance NK cell function in MM.

## MATERIALS AND METHODS

### Cell culture

Following cell lines KMS11, H929, RPMI8226, U266, K562 were purchased from ATCC (Manassas, VA, USA). OCI-MY5, OPM2, ARK, RPMI8226/R5 (MM-resistant cell line) were a kind gift from Professor Zhan (Department of Internal Medicine, University of Iowa, Iowa, USA). K562 was maintained in Iscove's Modified Dubecco's Medium (Gibco), supplemented with 10% fetal bovine-serum (Gibco) and 1% penicillin streptomycin–glutamine (Gibco). Other cells were grown in suspension in RPMI 1640 medium (Gibco) supplemented with 10% fetal bovine-serum (15% for U266) and 1% penicillin streptomycin–glutamine. All cells were incubated at 37°C in 5% carbon dioxide air atmosphere. CD138^+^ myeloma cells and CD56^+^/CD3^−^ NK cells were isolated from bone marrow and peripheral blood using magnetic bead selection (Miltenyi Biotech, Auburn, CA). Informed consent was obtained from each patient. All selected cells were more than 95% pure.

### Reagents and antibodies

CFZ was purchased from Onyx Pharmaceuticals. Monensin (Golgi-Stop, BD Biosciences) was used in the CD107a Degranulation Assay. Cell surface protein expression was determined by standard flow cytometry using unconjugated or fluorescein isothiocyanate (FITC)–, phycoerythrin (PE)–, and allophycocyanin (APC)–conjugated antibodies to CD56, CD107a, HLA-ABC, Major histocompatibility complex (MHC) class I chain-related (MIC) protein A (MICA) and B (MICB), UL16-binding proteins (ULBP1–3), annexin V, Mouse IgG_1_ (BD Pharmingen), rNKp30/human IgG1 Fc chimera, NKp44/human IgG1 Fc chimera, rNKp46/human IgG1 Fc chimera (R&D Systems), tumor necrosis factor related apoptosis-inducing ligand (TRAIL)-R1/DR4 and TRAIL-R2/DR5 (eBioscience). The concanamycin A (CMA; Abcam), anti-Fas-L (Abcam), anti-HLA class I ABC monoclonal antibody (mAb) (Abcam), anti-TRAIL mAb (eBioscience), NK receptor member D of the lectinlike receptor family (NKG2D) mAb, anti-hNKp30, anti-hNKp44, and anti-hNKp46 mAbs (R&D Systems) were used in the blocking experiments. Human β2M was purchased from Sigma and HLA-C antibody was purchased from Millipore. HLA-C binding peptides (EGDCAPEEK and RYPPVIVAY) were synthesized by Sangon Biotech.Co.Ltd (Shanghai, China). HLA typing showed that H929 was HLA-C0701, HLA-C0702. The first peptide was reported to bind with HLA-C07 [40], the second peptide was designed to bind with HLA-C0702 [41]. Cell apoptosis analysis kit (Annexin V-APC and 7AAD) was purchased from Nanjing KeyGEN Biotech.Co.Ltd (China).

### Flow cytometry

Cells were collected and washed once with phosphate-buffered saline (PBS). After that cells were stained with indicated unconjugated or FITC-, PE-, APC-conjugated antibodies or isotype antibody for 30 minutes at 4°C. Then cells were washed twice with PBS and stained with FITC-, APC-conjugated annexin V for 30 minutes at room temperature (RT), PI (BD) or 7AAD was added at last. Cells were analyzed after gating on PI (or 7AAD) negative or PI (or 7AAD) and annexin V double negative cells. Expressions were determined and mean-fluorescence intensity (MFI) was recorded on a flow cytometer (BD FASCCanto II). Class I decrease % = 100 × [MFI of control − MFI of treated cells] / MFI of control.

### Acid stripping and flow cytometry

H929 was used in the Acid treatment to remove HLA class I molecules from the cell surface. Cells were centrifuged and the resulting pellet was resuspended gently in 50 μl of 300 mM glycine (pH 2.5)/1% (w/v) BSA and incubated for 4 min at 37°C. The suspension was neutralized by dilution with 100 μl of cell medium containing 0.5 N NaOH and 0.2 M HEPES and centrifuged. Cells after acid stripping were resuspended in the presence or absence of 10 nM CFZ and incubated at 37°C for 12 hours to investigate the effect of CFZ on HLA class I re-expression.

### Immunofluorescence

H929 cell membranes were ruptured with 0.3% Triton X-100 (St. Louis, MO, USA) for 10 minutes at RT. After washed with ice-cold PBS once, H929 were labeled in suspension with FITC-anti HLA-ABC for 30 minutes at 4°C. Then cells were washed with ice-cold PBS once again. 2, 4-diamidino-2-phenylindole (DAPI) (1:1000) in PBS was used for staining nuclei. All sections were observed with fluorescence microscopy (Olympus).

### CD107a degranulation assay

CFZ treated MM cells were cocultured with NK cells at an effector/target (E/T) ratio of 5:1. FITC conjugated CD107a or isotype control was added in the beginning of the coculture. The culture condition was mentioned in Cell Culture. After 1 hour, monensin was added at the concentration of 6 μg /ml. Cells were incubated for another 3 hours. Then cells were collected and washed with PBS once and stained with PE-conjugated CD56 for 30 minutes at 4°C. After cells were washed with PBS once, 7AAD were added. Cells were analyzed on the flow cytometer and the data were analyzed using FCS Express 3 Flow Cytometry software.

### Cytotoxicity assay

^51^Cr release assay was used to analysis the NK cell-mediated lysis. Target cells were labeled with 100 μCi ^51^Cr (Perkin Elmer, Downers Grove, USA) for 1 h at 37°C. After that cells were washed with PBS three times to eliminate excess ^51^Cr and resuspended in 1ml medium. Then cells were cocultured with NK cells in triplicates at the indicated E/T ratio in the 96-well plate for 4 hours. The culture condition was mentioned in Cell Culture. The NK cell-sensitive cell line K562 was used as positive control. Specific lysis percentage was calculated as (experimental release − spontaneous release) / (maximal release − spontaneous release) × 100.

### Statistical analysis

The data are expressed as means ± standard deviations (SD). Differences among the experimental groups were determined with the Student's *t* test. All statistical analyses were performed with SPSS v20.0 statistical analysis software. Significance was established at a *p* value of 0.05 or less.

## SUPPLEMENTARY FIGURES


