# Supplementary material for: Carfilzomib enhances natural killer cell-mediated lysis of myeloma linked with decreasing expression of HLA class I
Source: Oncotarget. 2015 Aug 20;6(29):26982–94. doi: 10.18632/oncotarget.4831 (PMC4694968; doi:10.18632/oncotarget.4831)
Supplement: Supplementary file 1 [file oncotarget-06-26982-s001.pdf]

SUPPLEMENTARY FIGURES

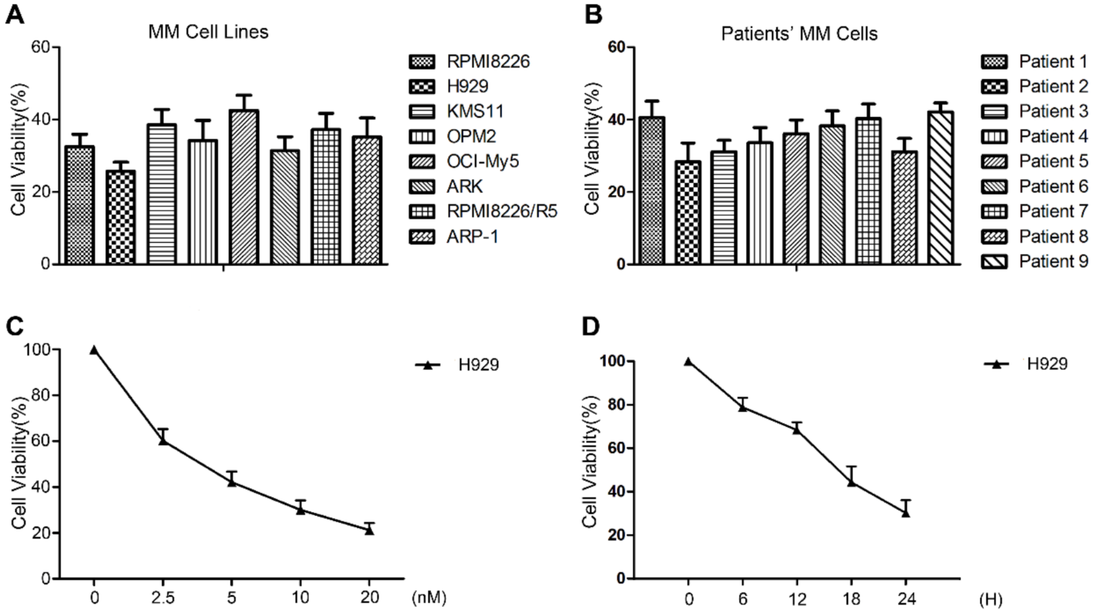

Supplementary Figure S1: CFZ induced apoptosis in MM cells. A. MM cell lines were treated with 10 nM Carfilzomib for 24 hours and stained with annexin V-FITC and 7AAD to detect apoptosis. **B.** Primary MM cells were treated with 20–40 nM Carfilzomib and stained with annexin V-FITC and 7AAD to detect apoptosis (20 nM: Patient 1–5, 7, 8; 40 nM: Patient 6, 9). **C.** A dose-dependent decrease in cell viability was found in H929 after CFZ treatment. **D.** A time-dependent decrease in cell viability was found in H929 after CFZ treatment.

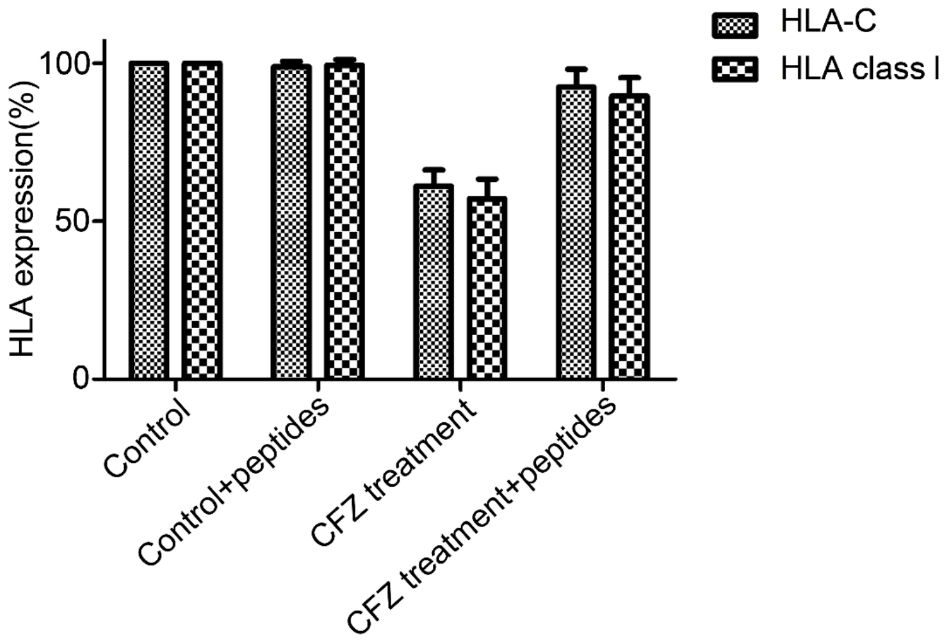

Supplementary Figure S2: Exogenous HLA-C binding peptides rescued the down-regulation of HLA-C and HLA-ABC caused by CFZ. H929 were treated with or without CFZ for 20 hours. HLA-C binding peptides (100 μg/ml) were added at the beginning and Human β2M (2 μg/ml) were added at the medium of the coculture. After 20 hours, cells were collected and flow cytometer was used to analyze the expression of HLA-C and HLA-ABC on cell surface.

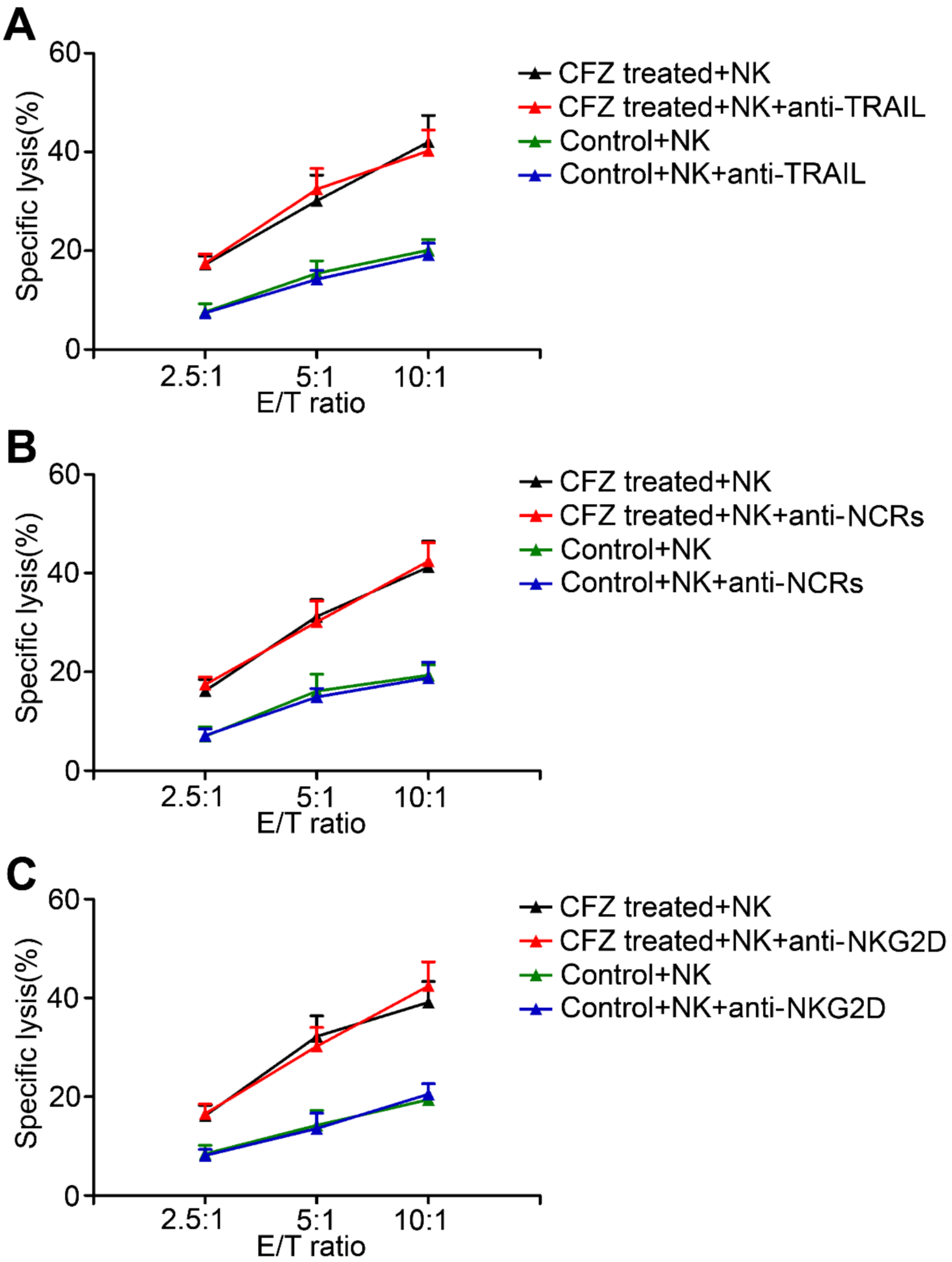

Supplementary Figure S3: Enhancement of NK cell-mediated lysis was associated with down-regulation of HLA class I. A. The enhancement of NK cell-mediated lysis was not mainly affected by the blocking of TRAIL on NK cells. **B.** The enhancement of NK cell-mediated lysis was not mainly affected by the blocking of NCRs on NK cells. **C.** The enhancement of NK cell-mediated lysis was not mainly affected by the blocking of NKG2D on NK cells. The E/T ratio were 2.5:1, 5:1, and 10:1.
